# Nanoscale Topography on Black Titanium Imparts Multi-biofunctional Properties for Orthopedic Applications

**DOI:** 10.1038/srep41118

**Published:** 2017-01-23

**Authors:** Jafar Hasan, Shubham Jain, Kaushik Chatterjee

**Affiliations:** 1 Department of Materials Engineering, Indian Institute of Science, Bangalore 560012, India

## Abstract

We have developed a chlorine based reactive ion etching process to yield randomly oriented anisotropic nanostructures that render the titanium metal surface ‘black’ similar to that of black silicon. The surface appears black due to the nanostructures in contrast to the conventional shiny surface of titanium. The nanostructures were found to kill bacteria on contact by mechanically rupturing the cells as has been observed previously on wings of certain insects. The etching was optimized to yield nanostructures of ≈1 μm height for maximal bactericidal efficiency without compromising cytocompatibility. Within 4 hours of contact with the black titanium surface, 95% ± 5% of *E. coli,* 98% ± 2% of *P. aeruginosa*, 92% ± 5% of *M. smegmatis* and 22% ± 8% of *S. aureus* cells that had attached were killed. The killing efficiency for the *S. aureus* increased to 76% ± 4% when the cells were allowed to adhere up to 24 hours. The black titanium supported the attachment and proliferation of human mesenchymal stem cells and augmented osteogenic lineage commitment *in vitro*. Thus, the bioinspired nanostructures on black titanium impart multi-biofunctional properties toward engineering the next-generation biomaterials for orthopedic implants.

Interactions with surface topographical features are widely known to affect the behavior of eukaryotic and prokaryotic cells on biomaterial surfaces^1^,^2^,^3^. These cell-surface interactions drive the biological response to materials and are critical in determining the success of biomedical implants *in vivo*. There have been several reports wherein topography mediated cell interactions with natural and bioinspired surfaces have been investigated for biomedical applications^4^,^5^,^6^,^7^,^8^,^9^. For example, microscale features on shark fins are known to resist bacterial attachment whereas the nanopillars on insect wings impart bactericidal activity to the surface^4^,^10^,^11^. Also, shark skin-inspired catheter surfaces showed reduced bacterial attachment and thrombosis^12^. Recently, black silicon was shown to exhibit bactericidal activity similar to insect wings^5^,^11^. For eukaryotic cells, bio-inspired anisotropic nanogrooves were more potent in inducing osteogenic and neurogenic differentiation of stem cells compared to grooves of other sizes^13^. Notably, many of these topographies have been prepared on model surfaces such as silicon wafers that do not find applications *in vivo*^5^,^11^. In addition to the bioinspired designs, few studies on topography mediated bactericidal activity and control of cell response on medically relevant materials have also been reported^14^,^15^. Osteogenic differentiation of mesenchymal stem cells was shown to be sensitive to nanoscale features embossed on polymethyl methacrylate^16^. In another study, osteogenic lineage commitment of stem cells was induced by titania nanotubes of a select size range^17^.

These strategies can offer significant benefits for designing multifunctional biomedical implants. Orthopedic devices such as hip joints, knee joints, bone plates, screws, etc. constitute some of the most widely used biomedical implants in the clinic with a global market worth billions of dollars^18^,^19^. Despite their widespread use, common limitations associated with their performance are bacterial infections and poor osseointegration *in vivo* that can lead to discomfort, pain and periimplantitis leading to repeat surgical procedures. There have been several incidences of hospital related bacterial infections caused by *Escherichia coli* and highly antibiotic resistant *Staphylococcus aureus* and *Pseudomonas aeruginosa* in post-operative surgeries, which require long term antibiotic therapies or periprosthetic replacements^20^,^21^,^22^,^23^,^24^. Moreover, the emergence of antibiotic resistant superbugs underscores the need for novel approaches for prevention and treatment of bacterial infections^25^. Thus, there is a need to design biofilm resistant surfaces preferably those that are bactericidal on contact. However, such bactericidal surfaces are not necessarily cytocompatible let alone osteogenic underscoring the need for novel approaches to design multi-biofunctional surfaces^5^,^26^. Interestingly, some natural surfaces are known to be bactericidal as well as cytocompatible^27^. Titanium and its alloys have emerged as the most promising class of materials for orthopedic implants owing to an excellent combination of desired properties such as mechanical properties, surface activity, corrosion resistance and inherent biocompatibility.

The objective of this work was to engineer a multi-biofunctional surface with nanoscale topography to impart bactericidal activity without loss in cytocompatibility and potentially augment osteogenic lineage commitment at the material-biology interface on titanium. A novel nanofabrication process of dry etching on a mask-less titanium metal is presented. The bactericidal activity and cytocompatibility of the engineered titanium surface was compared with that of the smooth surface. The ability of the surface to augment osteogenic lineage commitment of stem cells by the etched titanium surface was also assessed.

## Results and Discussion

The surface of commercially pure titanium samples was etched by RIE to generate nanoscale topography for maximal bactericidal activity and minimal cytotoxicity. Initially, the control titanium samples were etched for three different time intervals in order to determine the optimal time of etching for maximal biological response. The smooth titanium sample was etched for 5, 10 and 20 min. The photographs and micrographs of the smooth control and etched titanium samples are compiled in Electronic Supplementary Information, Figure S1. The surface nanostructures trap the light within and thus the characteristic shiny metallic appearance of the titanium surface turns black on all the etched samples. The effect is similar to the highly anisotropic etching effect on the black silicon surfaces^11^,^28^. The change in appearance of the etched titanium surface is analogous to that of the black silicon which is widely used in the semiconductor industry. The nanostructures lead to low reflectivity of black silicon and finds use in solar cells, photocathodes, and surface-enhanced Raman scattering (SERS) substrates as well as bactericidal materials more recently^29^,^30^,^31^. However, “black titanium” has not been reported and we propose it as a promising surface for biomedical applications, as further discussed below.

Etching of the titanium surface enhanced the surface water wettability (ESI, Figure S1). The surface roughness analysis confirmed the nanoscale smoothness of the control titanium surface prior to etching with mean squared average roughness value of 8 nm ± 1 nm (Table S1). The etched titanium surfaces were found to be rougher compared to the control due to the formation of random anisotropic nanostructures on etching. The height of the individual ‘nanopillars’ that constitute the nanostructures differed with the etching time as the etching rate was approximately 100 nm per minute. Due to the uniform presence of nanostructures over the surface, the effective surface area also increased with the etching time (SI, Table S1). The topography patterns of top regions of the control and etched surfaces showed that the profile changes with the etching time with formation of increasingly taller and more distinct peaks on the etched surfaces (SI, Figure S2).

To determine the optimal etching conditions to engineer a multi-biofunctional surface, we assessed the viability of bacterial and stem cells attached on the control and the nanostructured surfaces for three different etching times. The nanostructures formed by etching were found to mechanically rupture the *E. coli* cells. Staining revealed that the bacterial cell viability decreased with increase in the etching time reaching a saturating maximum at 10 min (SI, Figures S3 and S4). Mesenchymal stem cells are multipotent cells that can differentiate into bone among other lineages^32^. Measuring the viability of bone marrow-derived human mesenchymal stem cells (hMSCs) 3 days post seeding, it was observed that the fraction of viable cells was similar to the smooth control up to 10 min of etching but decreased significantly when titanium was etched for 20 min (SI, Figure S5). Thus, 10 min was taken as the optimal time of etching for engineering the multifunctional titanium surface for orthopedic applications with maximal bactericidal activity with minimal loss in cytocompatibility of titanium. This surface was used for further studies and is hereafter referred to as black titanium whereas the unetched smooth titanium surfaces will be referred to as the control titanium.

**Figure 1:**
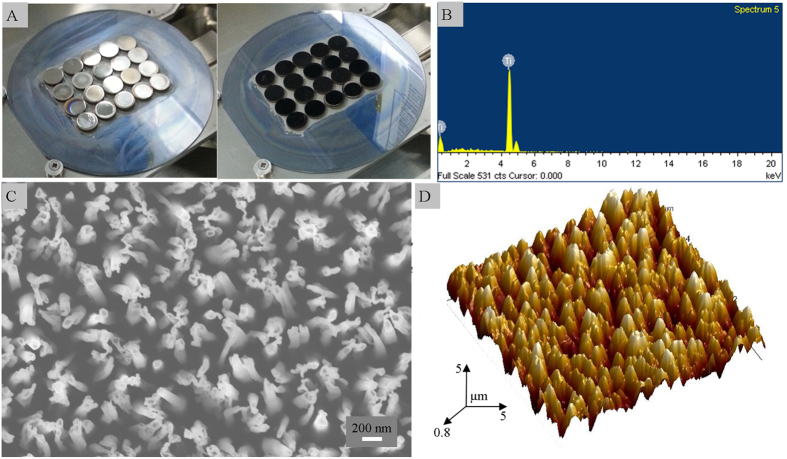
(**A**) Photographs of titanium discs before (left) and after (right) the etching process to prepare the black titanium. After etching, the smooth control titanium changes from a shiny metallic color to black due to the nanostructures that trap light. (**B**) EDS spectra of the black titanium surface. (**C**) SEM and (**D**) 3D AFM images of the black titanium surface showing random nanostructures.

Photographs of the control and black titanium discs before and after the etching, respectively, are shown in Fig. 1. Surface characterization of black titanium by energy dispersive spectroscopy (EDS) confirmed the presence of titanium as the major constituent of the nanostructured surface (Fig. 1B). The nanostructures are randomly oriented similar to the nanostructures present on black silicon and on insect wing surfaces (Fig. 1C)^4^,^11^. The height of the black titanium nanopillars was approximately 1 μm. The spacing between the nanopillars was random as many nanopillars were clustered together while the diameter of an individual nanopillar was ≈80 nm.

In order to characterize the chemical nature of the top layer and the inner regions of the nanostructures of black titanium, high resolution XPS spectra of Ti, O and Cl were recorded (Figure S6). The scans on the inner regions or the depth of the black titanium nanostructures were performed after Argon etching of 120 seconds. The comparison between the Ti spectra of the top layer of the surface and the depth of the black titanium shows the presence of a small shoulder of Ti^3+^2p_3/2_ on the top surface of the black titanium^33^. This additional shoulder is likely a result of the etching performed using chlorine gas on the top surface. Both the surface and the depth scans show characteristic Ti doublet peaks. The oxygen peaks typically represent the titanium oxide layer (O^2−^) which is present on the top layer as well as the depth of the titanium surfaces. The chlorine peaks are also found on the top layer and on the depth of the surface. However, the nature of the oxide and chloride detected on the inner regions of the surface maybe different from the nature of the oxide and chloride found on the top layer of the surface. The high resolution XPS spectra of Ti, O and Cl peaks of the smooth control titanium surface are presented in SI, Figure S7. Under the plasma conditions, the titanium is etched by a combination of chemical etching and ion bombardment. It has been reported that the chemical etching is primarily responsible for etching of the titanium metal whereas the oxide layer of titanium is removed during the ion bombardment step^34^. The characteristic XPS doublet of the titanium peak is indicative of the TiCl and TICl_2_ features^35^. It is reported that the chloride layer of titanium must be subjected to high energy ion bombardment as this step assists in chlorination^34^,^35^. Dry etching of titanium has earlier been reported with several patterned masks for biomedical applications^34^,^36^,^37^, but this is the first instance where an etching recipe has produced randomly oriented mask-less nanostructures generating a black titanium surface.

One of the advantages of titanium as a biomaterial is its excellent corrosion resistance among many other properties. Given the large increase in the surface area of the black titanium due to the nanopillars compared to that of the control, we assessed for possible change in the corrosion resistance of the metal using the potentiodynamic corrosion test in 0.9% NaCl solution. The corrosion rate for the control titanium surface determined from the Tafel plot was found to be 3.1 × 10^−6^ mA/cm^2^ whereas the corrosion rate for the black titanium was 6.3 × 10^−5^ mA/cm^2^ (Figure S8). Although the current density observed on the black titanium surface displayed one-fold increase over that of the control, both these values are lower than that of other metallic materials^38^,^39^. Thus, the black titanium exhibits good corrosion resistance and is well suited for use as a biomaterial.

**Figure 2:**
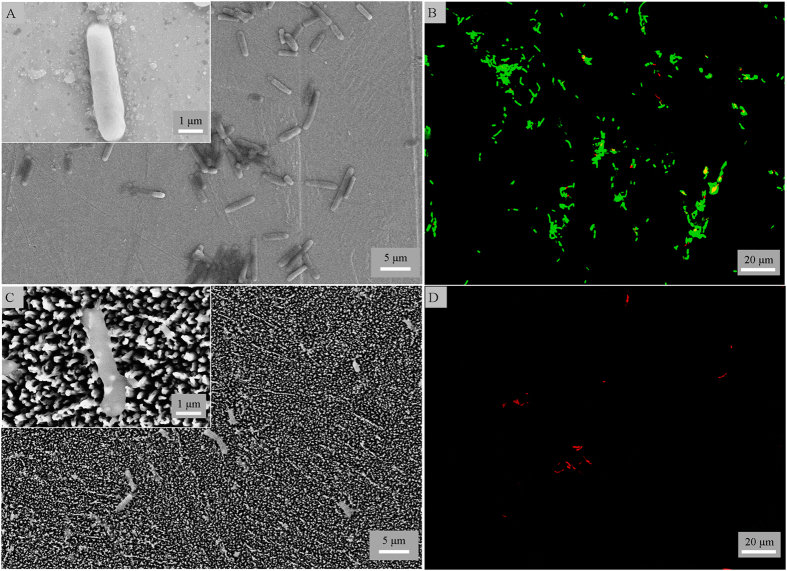
(**A**,**C**) SEM and (**B**,**D**) fluorescent microscopy images of *P. aeruginosa* attached for 4 hours on the control (top) and black titanium (bottom) surfaces.

**Figure 3:**
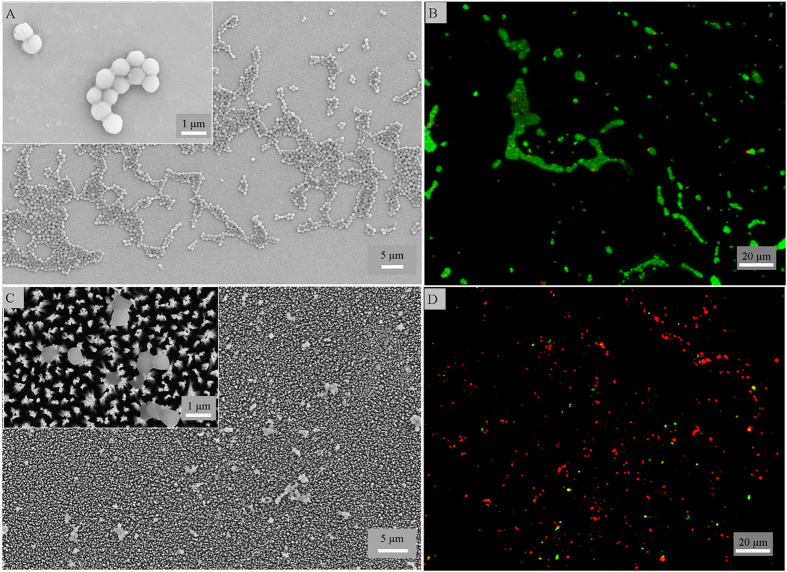
(**A**,**C**) SEM and (**B**,**D**) fluorescence micrographs of *S. aureus* attached for 24 hours on the control (top) and black titanium (bottom) surfaces.

A significant clinical challenge associated with biomedical implants is bacterial infections that commonly arise due to gram negative *P. aeruginosa* and gram positive *S. aureus* cells. Thus, we investigated the antibacterial activity with these cells to examine if the surfaces resist biofilm formation. The black titanium and the control surfaces were placed in suspensions of *P. aeruginosa*, and *S. aureus* cells for 4 hours. The morphology and viability of the attached cells was observed by SEM and fluorescent staining (Figs 2 and 3). The morphology of the bacterial cells in contact with the black titanium was significantly altered when compared to the morphology of the cells attached on the control. Quantitatively, the black titanium showed high bactericidal activity with killing efficiency of 98% ± 2% of the attached *P. aeruginosa* similar to the 95% ± 5% efficiency for the *E. coli* cells, as discussed above. These values are in sharp contrast to 6% ± 1% and 9% ± 4% efficiency on the control surface (Fig. 2 and SI, Figure S4). On surfaces tested with *S. aureus* cells, the black titanium exhibited a killing rate of 22% ± 8% in contrast to 3% ± 1% on the control surface. Since *S. aureus* cells are known to be prevalent in orthopedic implant and post-operative biofilm infections^40^,^41^, we examined if the black titanium surfaces are able inhibit the initiation of biofilm formation. The adhered cells were allowed to grow on the surface for additional 20 hours. It was observed that 76% ± 4% of the attached *S. aureus* cells are killed by the black titanium surface that is ≈10 times higher than the 7% ± 2% of the non-viable cells on the control surface (Fig. 3). To study the effect of black titanium on rod-shaped gram positive bacteria, we tested the attachment behaviour of *M. smegmatis*, a model species of the *Mycobacterium* genus. It was observed that 92% ± 5% of the *M. Smegmatis* attched on the black titaniuum were killed compared to the 9% ± 6% of non-viable cells on the control surface (Figure S9). Therefore, the black titanium surface exhibits excellent broad spectrum activity.

Overall, the highly efficient bactericidal behavior appears visibly similar to that reported for the insect wings where the bacterial cells look ruptured by the effect of the nanopillars (Figure S10). It is believed that the architecture of the nanopillars cause mechanical damage to the bacterial membrane, which has been widely reported recently for the insect wings as well as the black silicon surfaces^4^,^5^,^11^. However, such an enhanced bacterial killing behavior mediated by topography alone has been observed for the first time on a nanostructured titanium surface. It appears that the cells are stretched by the nanopillars similar to the mechanism reported in the case of cicada wing surfaces. Although the mechanism appears similar but it is to be noted that the geometry of the cicada wing surface is different from the nanoarchitecture of the black titanium. The growth of the representative gram negative (*E. coli*) and gram positive (*S. aureus*) bacterial cells on tissue culture plate, control titanium and black titanium samples was also assessed by measuring absorbance (Figure S11). It was found that the bacterial proliferation in the suspension on the black titanium samples is significantly attenuated when compared with the control titanium samples as the OD_600_ value on the black titanium samples is significantly lower than on the absorbance reading on the control titanium samples and the TCP plate.

Furthermore, the stability of the nanostructures of black titanium was assessed through prolonged sterilization procedures. Subjecting the black titanium discs to bath sonication for 3 hours followed by 20 cycles of high-pressure autoclaving did not alter the surface topography. The bactericidal activity of the black titanium surfaces was also retained confirming the functional stability of the surfaces.

From the literature, it is often observed that if the nanotopographical surface kills the bacterial cells then the eukaryotic cells are also compromised^5^,^26^. Alternatively, if the surface supports the attachment of eukaryotic cells, then the surface topography is only able to minimize the bacterial cell attachment at best and is thus anti-biofouling but does not show efficient bactericidal activity as observed here^8^,^42^,^43^,^44^. Huang *et al*., for example, studied the effect of hierarchical titanium surface topography that imparts a statistical decrease of adhered and proliferated bacterial cell numbers against the control surface. The nanoscale surface showed excellent antibiofouling property when compared with the micron/submicron-scale surface. Although the bacterial morphology appeared deformed bactericidal activity was not conclusively demonstrated. Hence, there is a large unmet need for designer surfaces that not only resist the relative attachment of bacterial cells but specifically damage them and yet support eukaryotic cell attachment and function for the next generation of biomedical devices. The black titanium presented here is not only demonstrated to be bactericidal for a wide variety of cells but did not compromise viability of mammalian cells (Figure S5).

**Figure 4:**
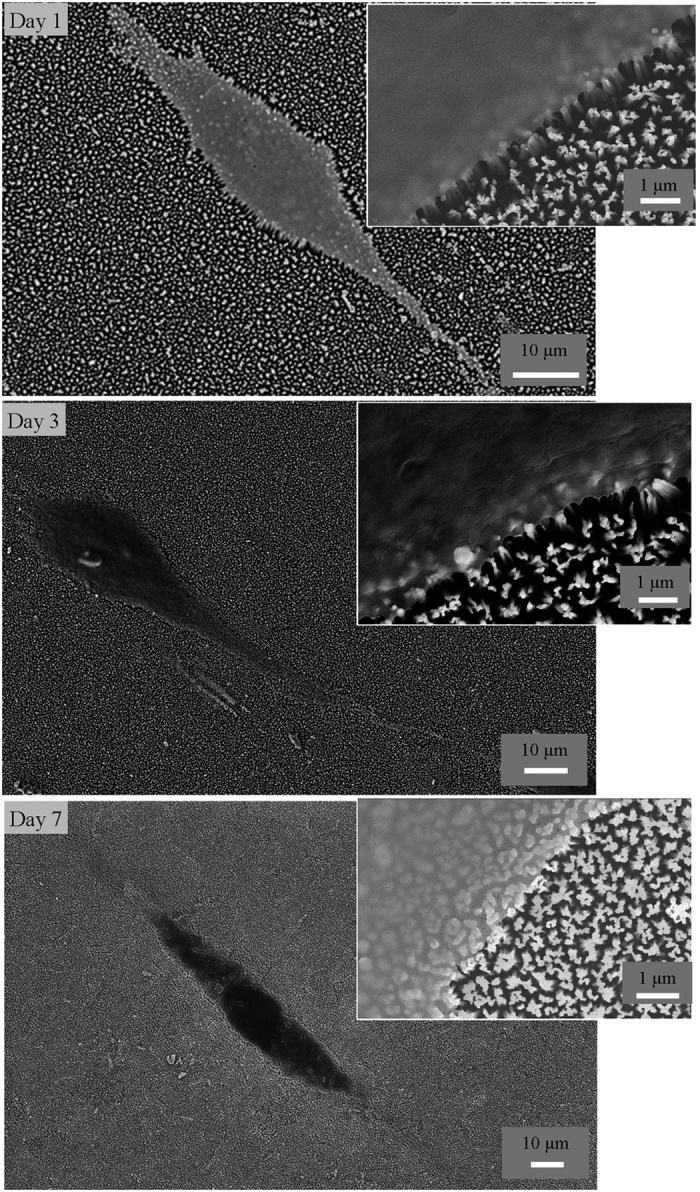
SEM images of hMSCs attached on the black titanium samples imaged at day 1, 3 and 7.

Given the good bactericidal activity, the black titanium surface was then evaluated its ability to support stem cell proliferation and possibly promote osteogenic lineage commitment for potentially fabricating orthopedic implants. Toward this aim, we studied the attachment and proliferation of hMSCs at 1, 3 and 7 days. It was seen that the hMSCs attached to the black titanium without discernable membrane damage as was observed for the bacterial cells. The nanostructures seem to serve as the anchorage points for the stem cells and the morphology of the cells appear to be well spread (Fig. 4). The morphology of the stem cells on the black titanium is markedly different in comparison to the attachment pattern on the control titanium (SI, Figure S12). The stem cells on the black titanium are slender and elongated compared to the equiaxed, spread cells observed on the control titanium surfaces.

**Figure 5:**
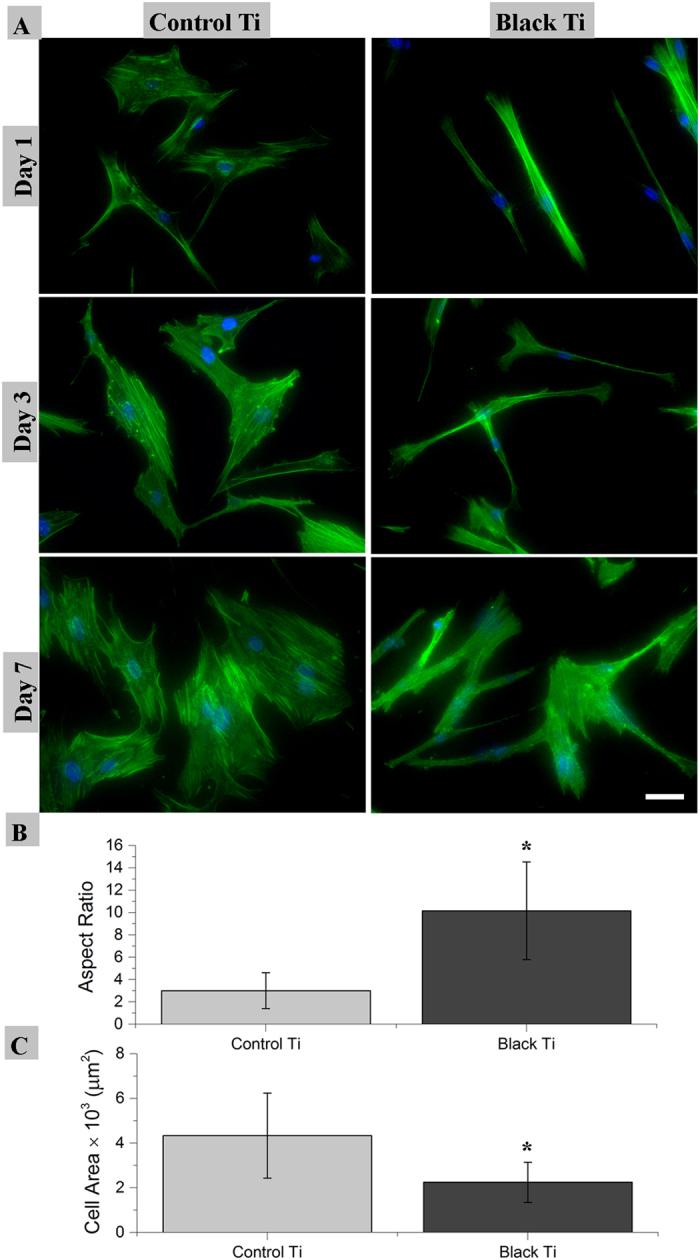
(**A**) Fluorescence micrographs (20x magnification) of hMSCs exhibiting F-actin (green) and nucleus (blue) attached on the control and black titanium samples over the period of 1, 3 and 7 days. Scale bar = 50 μm. Plots of the aspect-ratio (**B**) and spread area (**C**) of hMSCs on control and black titanium surfaces. Statistically significant difference (p < 0.05) is indicated by *.

**Figure 6:**
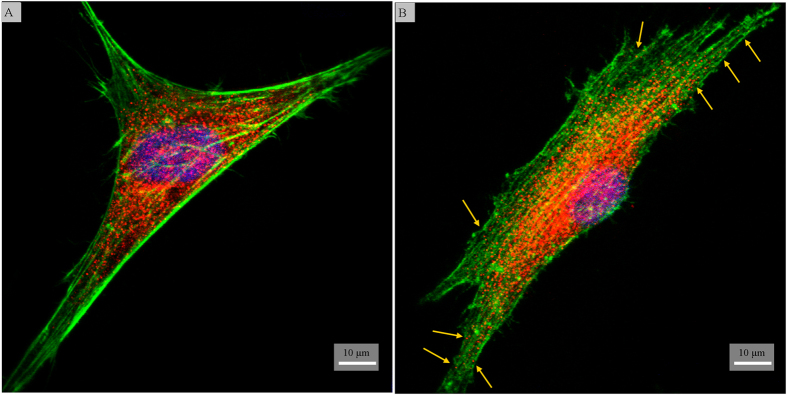
Confocal fluorescent micrographs of hMSCs stained for paxilin (red), actin filaments (green) and nucleus (blue) on control (**A**) and black (**B**) titanium surfaces.

**Figure 7:**
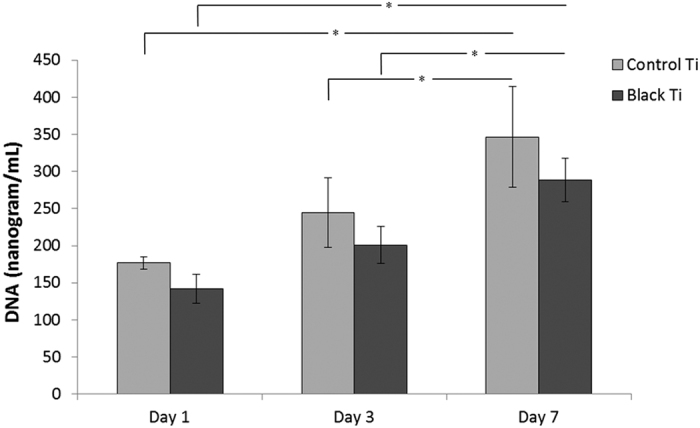
Plot of DNA content on the control and black titanium surfaces at day 1, 3 and 7. Statistically significant differences (p < 0.05) are indicated by the * symbol.

Cell nuclei and F-actin were stained to visualize cell morphology. The change in cellular morphology was quantified by analyzing micrographs of the fluorescently labeled cells (Fig. 5). hMSCs on the black titanium exhibited a statistically significant smaller spread area than on the control. The aspect ratio of the cells increased markedly on the black titanium consistent with the elongated morphology observed visually (Fig. 5). It has been shown that elongated cell morphology is an early indicator of osteogenic commitment^45^,^46^,^47^. The elongated morphology is thus envisaged to augment osteogenic lineage commitment of hMSCs on the black titanium surface. The adhesion of the cells on the titanium surfaces was revealed by the paxillin staining (Fig. 6). Both the black titanium and the control titanium surfaces support the attachment and proliferation of stem cells. The paxillin was observed in the center of the cells closer to the nucleus on the control, it was noticeably detectable at the outer edges on the black titanium surface (indicated by arrows). Such a distribution is characteristic of stronger attachment to the substrate and is believed to promote osteogenic commitment^48^,^49^. In order to quantify the cell numbers on both the substrates, the amount of dsDNA was measured using the PicoGreen assay (Fig. 7). The amount of DNA on the black titanium was marginally lower than that on the control titanium at 1, 3 and 7 days although these differences were not statistically significant. However, the amounts of DNA on the day 7 in comparison to the day 1 of both the control and titanium surfaces were significantly higher suggesting cell proliferation on both surfaces. Taken together, these data confirm that the black titanium substrate was not cytotoxic to the stem cells.

**Figure 8:**
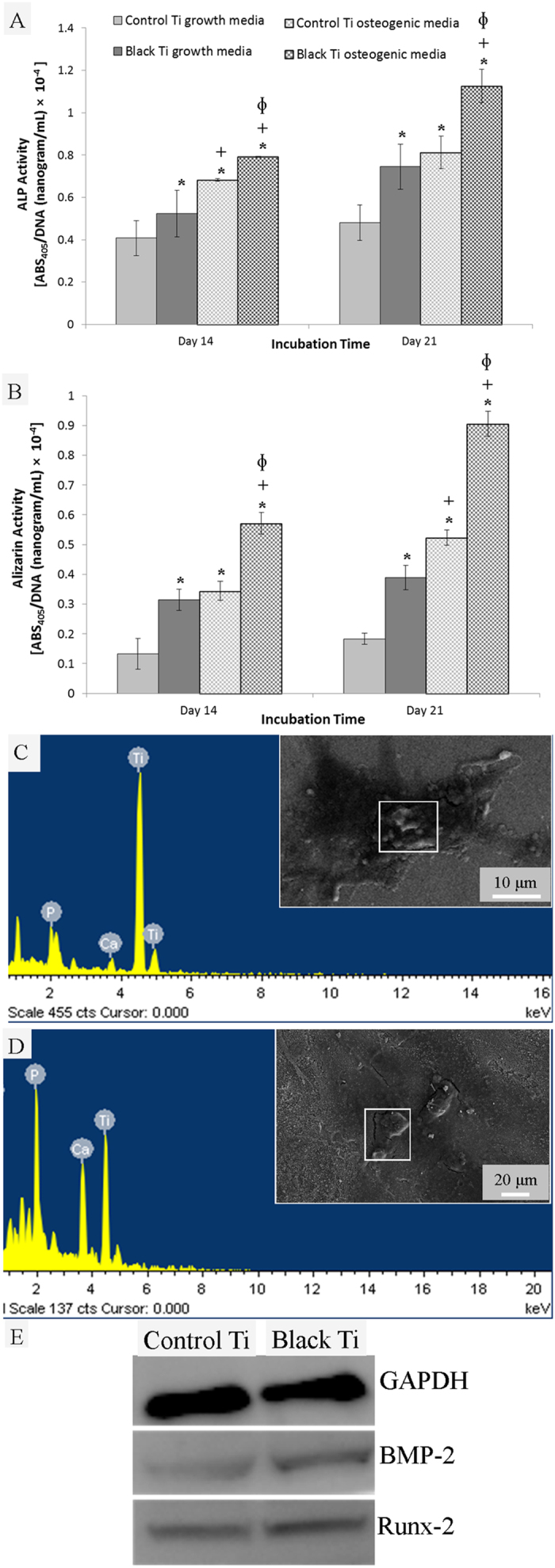
Plots of ALP expression (**A**) and mineral content (**B**) as markers of osteogenic commitment of hMSCs in growth and osteogenic media on the control and black titanium surfaces obtained after 14 and 21 days. Statistically significant differences (p < 0.05) compared to control Ti in growth media, black Ti in growth media and control Ti in osteogenic media are indicated by the symbols *, + and ɸ respectively. EDX spectra and corresponding SEM images of mineralized cells on the control (**C**) and black titanium (**D**) surfaces after 21 days showing the presence of calcium phosphate mineral deposits. (**E**) Relative protein expression assessed by Western blot technique (full blots shown in Figure S13).

We further assessed the osteoinductive property of black titanium in the presence of soluble factors (osteogenic medium) and also in growth medium. ALP expression was measured on day 14 and 21 (Fig. 8). ALP is taken as an early marker of osteogenic differentiation^50^. In osteogenic medium, ALP expression was significantly higher on the black titanium surface in comparison to the control surface at both 14 and 21 days. Not surprisingly, in the absence of the osteoinductive factors (growth medium), ALP expression was lower than in osteogenic medium on both surfaces. However, even in the growth medium, the expression was significantly higher on black titanium than the control. Thus, ALP expression data suggest that the black titanium augments osteogenic commitment of the cells.

Osteogenic differentiation eventually leads to the deposition of calcium phosphate. Thus, ARS staining was used to quantify the mineral deposition on the control and black titanium (Fig. 8). The measurements of the mineral content in osteogenic medium confirmed a significantly higher deposition on black titanium than the control titanium surfaces at both 14 and 21 days. The mineral content was markedly lower on both samples when growth medium was used. However, a similar trend was observed for growth medium where the mineral content was higher for the black titanium than the control on the both the days. EDS analysis confirmed the presence of calcium and phosphate on both the control and black titanium surfaces (Fig. 8). Interestingly, when normalized to the Ti peak, the Ca and P peaks were more intense for the black titanium than the control suggesting significantly higher mineral content on the black titanium.

To further confirm osteogenic commitment, the expression of some known markers was studied. BMP-2 is a bone morphogenic protein of the TGFβ superfamily and is a specific marker of skeletal development. It is known that BMP-2 signaling induces expression of Runx-2^51^. The change in protein expression was analyzed using Western blot (Fig. 8E and SI, Figure S13), which confirmed enhanced expression of BMP-2 and Runx-2 on the black titanium surface compared to the control surface. These results corroborate the results of ALP assay and mineral quantification.

Taken together, ALP measurements, mineral deposition and protein expression results confirm that the nanostructured black titanium augments osteogenic commitment of stem cells in both osteoinductive medium containing soluble factors and as well as in growth medium *in vitro*. Surface roughness plays an important role in osteogenic commitment and generally rougher surfaces (*R*_a_ values 0.2–1 μm) are known to promote differentiation^52^. The average roughness value of black titanium is comparable to the roughness values reviewed recently^52^. Other parameters of roughness must also be taken into account especially in highly random nanopillar surfaces. Due to the complexity of the randomly oriented nanopillars, the geometric dimensions that elicit optimum osteogenic commitment have not been categorized. However, it is generally agreed upon that introduction of nanopillars enhances the osteogenic commitment of cells^53^,^54^, particularly on disordered nanotopographies^16^. The preferential proliferation and osteogenic commitment of the stem cells with excellent resistance to bacterial attachment on the black titanium surface is a unique and novel surface property. Such multi-biofunctional materials are a key to designing the next generation of biomedical implants.

Since the recent discovery of the bactericidal insect wings exhibiting nanopillars^4^,^11^, the phenomena underlying the killing of the bacterial cells is just starting to be understood. Broadly, the mechanical rupturing of the bacterial cells by the black titanium seems to result from the stretching of the bacterial membrane by the nanostructures. Several mechanical factors such as the spacing between the nanopillars, the width of the nanopillars, the cell shape and the cell membrane rigidity also play significant roles in the attachment behavior. This has been shown by several models where similar surface topography is seen to damage the bacterial cells^55^,^56^,^57^. The selective behavior of the nanostructures of the black titanium against bacterial and eukaryotic cells is reported here as a phenomenon that requires detailed analysis for developing a comprehensive analysis.

There are several differences between the mammalian and bacterial cells that influenced the interactions with the nanopillars on black titanium. The differential stiffness of the eukaryotic and bacterial cells is one plausible mechanism. It has been reported that the Young’s modulus of stem cells is in the range of 0.09 to 50 kPa whereas that of bacterial cell envelopes are three orders of magnitude higher in the range of 50 to 200 MPa^58^,^59^. It appears that the relatively high modulus of the bacterial cell provides enhanced rigidity and perhaps the resistance against the highly stiff nanostructures of the black titanium eventually leads to its rupture. In contrast, the much lower modulus of the hMSCs possibly imparts sufficient flexibility to perhaps conform to the surface topography without rupturing. Furthermore, the nature of cell-substrate interactions is fundamentally different for mammalian and bacterial cells that may contribute to the observed differences. Mammalian cells actively sense and respond to surface topography through modulation of their morphology mediated by the cytoskeleton and formation of focal adhesions. The bacterial cells do not adapt to such topographical features with a more static morphology such that the nanopillar are perhaps more efficiently able to disrupt the membrane and compromise its integrity. Qualitatively, it seems that the interaction of the bacterial cells with the black titanium surface is similar to the one observed and explained in case of the cicada wing surface^4^,^55^. However, the geometric architecture of the black titanium nanopillars is significantly different and therefore, newer mathematical models must be developed that can describe the selective phenomena that imparts the unique bactericidal, cytocompatible and osteogenic properties to black titanium. Moreover, comprehensive *in vivo* tests are warranted to demonstrate the enhanced osteogenic activity of black titanium in addition to its ability to minimize infections for clinical use. Interestingly, biomedical implants that contact the bone tissue have shown increased success *in vivo* when the contacting surfaces possess rough morphology to facilitate enhanced osseointegration^60^,^61^,^62^,^63^. It has been shown that macroporous Ti implanted in dog models assists in osteoinduction^60^.

## Conclusion

In summary, anisotropic nanostructures were fabricated to yield black titanium by chlorine based reactive ion etching. Subsequently, the topography, wettability and chemical composition of the surface were characterized. The *R*_q_ value of the black titanium surface was 227 ± 8 nm which was significantly rougher than the 8 ± 1 nm of the control titanium surface. Contact angle goniometry revealed that the black titanium surface showed higher water wettability than the control surface. The XPS spectra showed the presence of Ti and Cl on the black titanium surface due to the chlorine etching step. The nanoscale topography killed a wide variety of bacterial cells on contact in a manner similar to that of insect wings and black silicon. Black titanium supported attachment and proliferation of hMSCs. It was further observed that the black titanium augmented osteogenic commitment. These findings suggest that such a black titanium surface with nanoscale features could be well suited for engineering the next generation orthopedic implants with multi-biofunctional properties.

## Methods

### Titanium surface preparation

Commercially pure titanium plates (grade 2) were used for this study. The titanium plates of 3 mm thickness were cut to circular discs of 10 mm diameter by electrical discharge machining. The titanium discs were then polished with silicon carbide abrasive paper of varying grade sizes from 320 to 4000 followed by standard cloth polishing. The polished titanium discs were used for etching and also served as the smooth control.

### Reactive ion etching

Chlorine based reactive ion etching was performed using Oxford Instruments (Plasmalab System 100) to etch the titanium discs. The chlorine gas flow was maintained at 30 sccm. The RF power for ICP and RF generators were 1000 W and 50 W respectively. The chamber pressure was kept at 3 mTorr. The etch rate was 100 nm per minute. Etching was performed for 5, 10 and 20 minutes to fabricate three different etched titanium surfaces. The unetched control and black titanium surfaces were cleaned in IPA, acetone and ethanol prior to characterization and biological study.

### Physico-chemical characterization

The static contact angle of ultrapure water (Sartorius Arium) was measured using a contact angle goniometer (OCA 15EC, Dataphysics) on the control and black titanium surfaces. The image of the water droplet was taken 1 second after dispensing 1 μL of water on the sample. The contact angle was measured using the ImageJ software. Three independent replicates were used for each sample. Surface morphology of the control and black titanium were observed using a scanning electron microscope (SEM, Ultra55, Gemini) set at 7 kV with a secondary or in-lens detector. The EDX spectra were obtained on the titanium surface at 15 kV using X-ray spectrometer (INCA suite v 4.15) interfaced with the SEM.

Surface topography of the top regions of the control and black titanium was evaluated using an atomic force microscope (Bruker, Dimension Icon ScanAsyst) in tapping mode at the room temperature. A cantilever of Tespa model (Bruker) with spring constant of 20–80 N/m and frequency of 273–377 kHz was used.

The titanium, oxygen and chlorine content of the surfaces were characterized using X-ray photoelectron spectroscopy (XPS, Axis Ultra). High resolution XPS spectra were recorded using a monochromatic Al source (1.486 keV, Kratos Analytical) at the outermost surface and after ion etching. Samples were etched with Ar for 120 s to record XPS data at depth.

### Corrosion response

Corrosion behavior of the control and black titanium surface was measured in 0.9% of NaCl solution as reported earlier^64^. A three-electrode potentiostat (CH Instruments) was used with platinum as a counter electrode and Ag/AgCl electrode as a reference electrode. Samples were immersed in 0.9% of NaCl for 3 hours for the Open Circuit Potential (OCP) to stabilize. Subsequently, polarization was performed from −600 mV to +600 mV at a rate of 12 mV min^−1^. Corrosion potential (E) and corrosion current density (I) were calculated by the Tafel extrapolation method.

### Bacterial studies

Gram negative bacterial strains of *Escherichia coli* (ATCC 25922), *Pseudomonas aeruginosa* (ATCC 27853) and gram positive strains of *Staphylococcus aureus* (ATCC 25923) and *Mycobacterium smegmatis* (MC255) were used. *M. smegmatis* was grown in 7H9 Middlebook broth with 0.2% of glycerol and 0.05% of Tween 80. *M. smegmatis* was grown overnight at 250 rpm and 37 °C. All other bacterial strains were grown in 100 mL of sterile nutrient broth (HiMedia) overnight at 200 rpm and 37 °C. Bacterial cultures were cultured on nutrient agar (HiMedia). All the cells were collected at the logarithmic stage of growth and the concentration of the suspensions was adjusted to OD_600_ (optical density at 600 nm) value of 0.10–0.15 in the nutrient broth solution. The smooth control and etched titanium discs of diameter 10 mm were immersed in 200 μL of the bacterial suspension in a 48-well tissue culture polystyrene (TCPS) plate. The surfaces were incubated in 48-well plate with *E. coli, P. aeruginosa, S. aureus* and *M. smegmatis* cells for 4 hours at 37 °C in the nutrient broth before imaging. Additionally, the surface were incubated with the gram-positive *S. aureus* cells for 24 hours where the nutrient broth was removed after 4 hours and fresh nutrient broth was added to study the effect of bacterial growth of the cells in contact with the surface. To assess the morphology of the adhered bacterial cells, the control and etched titanium surfaces were washed with fresh PBS and the cells were fixed with 2.5% glutaraldehyde for 30 minutes. The samples were sputtered coat with gold prior to imaging by the SEM.

Viability of the adhered bacterial cells was determined by the cells stained with the LIVE/DEAD BacLight Bacterial Viability kit (Molecular Probes, Invitrogen). Adherent cells were stained using 3.3 mM SYTO 9 and 20 mM Propidium iodide for 15 min and imaged with a fluorescence microscope (Olympus) at 40x magnification in the green and red channels for the live and dead cells, respectively. The fraction of viable cells was determined by counting cells stained both green and red from fifteen images of at least three independent replicates. The proliferation of two strains, namely *E. coli* and *S. aureus* was also evaluated by measuring the OD_600_ of the bacterial cells at 0, 4 and 24 hour time interval suspended in nutrient broth solution. The measurements were performed on suspensions with OD_600_ value starting from 0.1 on control and black titanium samples kept in a 48 well plate. The absorbance readings were also measured on the 48 well TCP plate without any titanium samples.

### Stem cell viability and proliferation

All experiments were conducted with permission from the Institutional Committee on Stem Cell Research. Primary bone-marrow derived human mesenchymal stem cells (hMSCs) from a 25 year old male patient were procured commercially (Stempeutics, India) and cultured in complete culture medium prepared from knockout Dulbecco’s Modified Eagle Medium (DMEM, Invitrogen), as reported earlier^65^. The medium was supplemented with 1% glutamax, and 1% penicillin−streptomycin antibiotic mixture (Sigma) and 15 vol % MSC-qualified fetal bovine serum (FBS, Himedia). The culture flask was incubated at 5% CO_2_ atmosphere at 37 °C. Medium was changed every three days until 80% confluence was reached. Cells were suspended using 0.25% trypsin (Gibco) and counted using a haemocytometer. All the control and etched titanium surfaces were sterilized by immersing in ethanol for 30 min followed by exposure to UV for 1 h prior to seeding the cells. Titanium discs were placed individually in a 48-well plate. 400 μL of medium containing 5000 cells was added to each well.

For the viability analysis, the LIVE/DEAD^®^ Viability/Cytotoxicity Assay Kit (Molecular Probes, Invitrogen) was utilized. Adherent cells were stained using 2 μM Calcein and 4 μM Ethidium homodimer-1 at 37 °C for 15 minutes in order to determine the fraction of viable cells from the fluorescence images.

Cell attachment and proliferation were analyzed by staining the actin filaments and nuclei of the hMSCs on 1, 3 and 7 days after seeding. The fluorescently labeled cells were imaged to characterize cell morphology. Six replicates of each sample were used: three replicates for SEM and three for fluorescent imaging. For fluorescence imaging, cells were fixed using 3.7% formaldehyde at 37 °C for 15 min. The cells were subsequently permeabilized with 0.2% Triton X (Sigma Aldrich). Actin filaments were stained using 25 μg/mL Alexa Fluor 488 (Invitrogen) at 37 °C for 15 min. Cell nuclei were stained using 0.2 μg/mL DAPI (Invitrogen) at 37 °C for 5 min. Stained cells were imaged with an inverted fluorescence microscope (Olympus) at 4x, 10x and 20x magnification. For SEM imaging, adherent cells on the control and black titanium surfaces were washed with PBS solution and fixed with 3.7% formaldehyde at 37 °C for 15 min. The samples were then mounted and gold sputtered prior to SEM imaging.

To visualize the focal adhesion of cells, paxillin staining was performed after cell fixation and permeabilization. 0.2% of fish skin gelatin in PBS and 0.02% of Tween were used for blocking for 45 minutes at 25 °C. The cells were then incubated with the primary antibody (Paxillin, Abcam 2264) and further diluted (1:200) with the blocking buffer overnight at 4 °C. Anti-rabbit secondary antibody conjugated with Cy3 (red) was used. Cells were then incubated with the secondary antibody for 45 minutes at room temperature. The nuclei and actin filaments were stained with DAPI (blue) and Alexa Fluor 488 (green) for 5 and 30 minutes, respectively. Finally, the cells were imaged using the confocal microscope (Leica SP5).

The cell area and aspect ratio of hMSCs were calculated from thirty random fluorescent images using the ImageJ software for at least as many individual cells on the control and black titanium samples at day 3.

Attachment and proliferation were further characterized using quantification of cell numbers. The cell numbers on the films were determined by measuring the DNA content on each surface. The Picogreen dsDNA Quantitation kit (Molecular Probes) was used in order to quantify the DNA content of the hMSCs attached on the control and black titanium surfaces. For this, the cells were initially lysed using 200 μL of lysis solution (0.2 mg/mL of proteinase K (Sigma) and 0.02% of sodium dodecyl sulfate (Sigma)). Samples were incubated at 37 °C for 24 hours. 100 μL of the lysate solution was mixed with 100 μL of the Picogreen working solution. Fluorescence intensity of the solutions was measured using a microplate reader (Biotek, USA) at 485 nm excitation and 528 nm emission wavelength. Six independent samples were used to quantify the DNA content.

### Stem cell differentiation

Osteogenic commitment of the stem cells was assessed for cells cultured in growth medium and osteoinductive medium (growth medium supplemented with osteoinductive factors including 10 nM dexamethasone, 20 mM β-glycerophosphate and 50 μM ascorbic acid, all obtained from Sigma). To assess differentiation induced by the titanium surfaces, alkaline phosphatase (ALP) expression and mineral deposition staining were assayed on the 14^th^ and 21^st^ day post seeding. To quantify the ALP expression and mineral content, the data were normalized to the cell numbers (DNA content) quantified by the Picogreen assay on day 14 and 21. ALP expression was measured using the p-nitrophenyl phosphate (pNPP Sigma). Cells were lysed with 0.1% of Triton X −100 for 24 hours and then freeze–thawed from −80 to 37 °C for 10 min. After thawing, 100 μL of lysate was collected in a 96 well plate and an equal amount of p-nitrophenyl phosphate was added. The absorbance reading at 405 nm was measured after 1 hour of incubation using a microplate reader (Biotek). To assess the amount of calcium mineral deposited, the medium was first aspirated and the titanium surfaces were washed with distilled water. The cells were fixed with 3.7% formaldehyde solution for 30 minutes. Mineral content was quantified with the Alizarin red-S dye (ARS, Sigma). Fixed cells were stained with 1% of ARS dye for 15 minutes. The unbound dye was removed carefully by washing in distilled water. Stained ARS was dissolved in 0.2 mL of 5% SDS in 0.5 N HCl for 30 minutes. Subsequently, the absorbance of the solubilized dye was measured at 405 nm using a microplate reader. Also, the mineralized titanium surfaces were imaged using the SEM and the chemical nature of the mineral deposited on the surfaces was analyzed by SEM interfaced energy dispersive X-ray spectroscopy (EDS) at 15 kV using the X-ray spectrometer (INCA suite v 4.15).

The expression of proteins Runx-2 and BMP-2 (bone morphogenic protein) with GAPDH as the internal house-keeping control by hMSCs in growth medium was assessed by the Western blot technique on day 14 on both the control and black titanium surfaces. Respective primary anti-human antibodies raised in goat and secondary anti-goat antibody raised in rabbit were commercially obtained (Pierce Antibodies, Thermoscientific). The blots were developed and analyzed for chemiluminescence using the myECL imager (Pierce, Thermoscientific).

### Statistical analysis

Statistically significant differences among the different samples were analyzed using 1-way ANOVA (analysis of variance) with Tukey’s test for multiple comparisons. Differences were considered statistically significant for p < 0.05 and indicated by various symbols in the respective figures.

## Additional Information

**How to cite this article**: Hasan, J. *et al*. Nanoscale Topography on Black Titanium Imparts Multi-biofunctional Properties for Orthopedic Applications. *Sci. Rep.* **7**, 41118; doi: 10.1038/srep41118 (2017).

**Publisher's note:** Springer Nature remains neutral with regard to jurisdictional claims in published maps and institutional affiliations.

## Supplementary Material

Supplementary Information

## References

[b1] Park J. . TiO2 Nanotube Surfaces: 15 nm—An Optimal Length Scale of Surface Topography for Cell Adhesion and Differentiation. Small 5, 666–671 (2009). 19235196 10.1002/smll.200801476

[b2] Jeon H. . Directing cell migration and organization via nanocrater-patterned cell-repellent interfaces. Nat. Mater. 14, 918–923 (2015). 26213899 10.1038/nmat4342 PMC4545687

[b3] Hasan J. & Chatterjee K. Recent advances in engineering topography mediated antibacterial surfaces. Nanoscale 7, 15568–15575 (2015). 26372264 10.1039/c5nr04156b PMC4642214

[b4] Ivanova E. P. . Natural bactericidal surfaces: mechanical rupture of *Pseudomonas aeruginosa* cells by cicada wings. Small 8, 2489–2494 (2012). 22674670 10.1002/smll.201200528

[b5] Hasan J., Raj S., Yadav L. & Chatterjee K. Engineering a nanostructured “super surface” with superhydrophobic and superkilling properties. RSC Adv. 5, 44953–44959 (2015). 10.1039/C5RA05206H PMC5654505 29075481

[b6] Unadkat H. V. . An algorithm-based topographical biomaterials library to instruct cell fate. Proc. Natl. Acad. Sci. 108, 16565–16570 (2011). 21949368 10.1073/pnas.1109861108 PMC3189082

[b7] Hasan J., Crawford R. J. & Ivanova E. P. Antibacterial surfaces: the quest for a new generation of biomaterials. Trends Biotechnol. 31, 295–304 (2013). 23434154 10.1016/j.tibtech.2013.01.017

[b8] Bhadra C. M. . Antibacterial titanium nano-patterned arrays inspired by dragonfly wings. Sci. Rep. 5, 16817 (2015). 26576662 10.1038/srep16817 PMC4649496

[b9] Fisher O. Z., Khademhosseini A., Langer R. & Peppas N. A. Bioinspired Materials for Controlling Stem Cell Fate. Acc. Chem. Res. 43, 419–428 (2010). 20043634 10.1021/ar900226q PMC2840210

[b10] Chung K. K. . Impact of engineered surface microtopography on biofilm formation of Staphylococcus aureus. Biointerphases 2, 89–94 (2007). 20408641 10.1116/1.2751405

[b11] Ivanova E. P. . Bactericidal activity of black silicon. Nat. Commun. 4 (2013). 10.1038/ncomms3838 PMC3868328 24281410

[b12] May R. M. . An engineered micropattern to reduce bacterial colonization, platelet adhesion and fibrin sheath formation for improved biocompatibility of central venous catheters. Clin. Transl. Med. 4, 9 (2015). 25852825 10.1186/s40169-015-0050-9 PMC4385044

[b13] Kim J. . Designing nanotopographical density of extracellular matrix for controlled morphology and function of human mesenchymal stem cells. Sci. Rep. 3, 3552 (2013). 24352057 10.1038/srep03552 PMC6506445

[b14] Dalby M. J., Gadegaard N. & Oreffo R. O. C. Harnessing nanotopography and integrin-matrix interactions to influence stem cell fate. Nat. Mater. 13, 558–569 (2014). 24845995 10.1038/nmat3980

[b15] Dickson M. N., Liang E. I., Rodriguez L. A., Vollereaux N. & Yee A. F. Nanopatterned polymer surfaces with bactericidal properties. Biointerphases 10, 021010 (2015). 26077558 10.1116/1.4922157 PMC4474951

[b16] Dalby M. J. . The control of human mesenchymal cell differentiation using nanoscale symmetry and disorder. Nat. Mater. 6, 997–1003 (2007). 17891143 10.1038/nmat2013

[b17] Park J., Bauer S., von der Mark K. & Schmuki P. Nanosize and Vitality: TiO2 Nanotube Diameter Directs Cell Fate. Nano Letters 7, 1686–1691 (2007). 17503870 10.1021/nl070678d

[b18] Suter L. G. . Placing a Price on Medical Device Innovation: The Example of Total Knee Arthroplasty. PloS one 8, e62709 (2013). 23671626 10.1371/journal.pone.0062709 PMC3646021

[b19] Schwarzkopf R. . Factors Influencing Patients’ Willingness to Pay for New Technologies in Hip and Knee Implants. J. Arthroplasty 28, 390–394 (2013). 23142436 10.1016/j.arth.2012.07.009

[b20] Zimmerli W., Trampuz A. & Ochsner P. E. Prosthetic-Joint Infections. N. Engl. J. Med. 351, 1645–1654 (2004). 15483283 10.1056/NEJMra040181

[b21] Sendi P. . *Escherichia coli* Variants in Periprosthetic Joint Infection: Diagnostic Challenges with Sessile Bacteria and Sonication. J. Clin. Microbiol. 48, 1720–1725 (2010). 20335421 10.1128/JCM.01562-09 PMC2863887

[b22] Gellatly S. L. & Hancock R. E. W. Pseudomonas aeruginosa: new insights into pathogenesis and host defenses. Pathog. Dis. 67, 159–173 (2013). 23620179 10.1111/2049-632X.12033

[b23] Brouqui P., Rousseau M. C., Stein A., Drancourt M. & Raoult D. Treatment of Pseudomonas aeruginosa-infected orthopedic prostheses with ceftazidime-ciprofloxacin antibiotic combination. Antimicrob. Agents Chemother. 39, 2423–2425 (1995). 8585720 10.1128/aac.39.11.2423 PMC162959

[b24] Liu C. . Clinical Practice Guidelines by the Infectious Diseases Society of America for the Treatment of Methicillin-Resistant Staphylococcus aureus Infections in Adults and Children. Clin. Infect. Dis. 52, e18–e55 (2011). 21208910 10.1093/cid/ciq146

[b25] Arias C. A. & Murray B. E. Antibiotic-resistant bugs in the 21st century—a clinical super-challenge. N. Engl. J. Med. 360, 439–443 (2009). 19179312 10.1056/NEJMp0804651

[b26] Pham V. T. H. . Nanotopography as a trigger for the microscale, autogenous and passive lysis of erythrocytes. J. Mater. Chem. B 2, 2819–2826 (2014). 10.1039/c4tb00239c 32261476

[b27] Watson G. S. . A gecko skin micro/nano structure – A low adhesion, superhydrophobic, anti-wetting, self-cleaning, biocompatible, antibacterial surface. Acta Biomater. 21, 109–122 (2015). 25772496 10.1016/j.actbio.2015.03.007

[b28] Jansen H., de Boer M., Legtenberg R. & Elwenspoek M. The black silicon method: a universal method for determining the parameter setting of a fluorine-based reactive ion etcher in deep silicon trench etching with profile control. J. Micromech. Microeng. 5, 115 (1995).

[b29] Seniutinas G. . Versatile SERS sensing based on black silicon. Optics express 23, 6763–6772 (2015). 25836894 10.1364/OE.23.006763

[b30] Liu X. . Black silicon: fabrication methods, properties and solar energy applications. Energy & Environmental Science 7, 3223–3263 (2014).

[b31] Shen L. . Nanostructured Silicon Photocathodes for Solar Water Splitting Patterned by the Self-Assembly of Lamellar Block Copolymers. ACS Appl. Mater. Interfaces 7, 26043–26049 (2015). 26575400 10.1021/acsami.5b08661

[b32] Pittenger M. F. . Multilineage Potential of Adult Human Mesenchymal Stem Cells. Science 284, 143–147 (1999). 10102814 10.1126/science.284.5411.143

[b33] Hebenstreit E. L. D. . The adsorption of chlorine on TiO2(1 1 0) studied with scanning tunneling microscopy and photoemission spectroscopy. Surf. Sci. 505, 336–348 (2002).

[b34] Parker E., Thibeault B., Aimi M., Rao M. & MacDonald N. Inductively coupled plasma etching of bulk titanium for MEMS applications. J. Electrochem. Soc. 152, C675–C683 (2005).

[b35] Fracassi F. & d’Agostino R. Chemistry of titanium dry etching in fluorinated and chlorinated gases. Pure Appl. Chem. 64, 703–707 (1992).

[b36] Gott S. C., Jabola B. A. & Rao M. P. Vascular stents with submicrometer-scale surface patterning realized via titanium deep reactive ion etching. J. Micromech. Microeng. 25, 085016 (2015).

[b37] Vandrangi P., Gott S. C., Kozaka R., Rodgers V. G. & Rao M. P. Comparative endothelial cell response on topographically patterned titanium and silicon substrates with micrometer to sub-micrometer feature sizes. PloS one 9, e111465 (2014). 25357245 10.1371/journal.pone.0111465 PMC4214724

[b38] Ameer M. A., Khamis & Al-Senani. Adsorption Studies of the Effect of Thiosemicarbazides on the Corrosion of Steel in Phosphoric Acid. Adsorpt. Sci. Technol. 18, 177–194 (2000).

[b39] Saito M. . Corrosion Properties of Electroplated CoNiFe Films. J. Electrochem. Soc. 146, 2845–2848 (1999).

[b40] Singhal D., Foreman A., Bardy J. J. & Wormald P. J. Staphylococcus aureus biofilms. The Laryngoscope 121, 1578–1583 (2011). 21647904 10.1002/lary.21805

[b41] Harris L. G. & Richards R. G. Staphylococci and implant surfaces: a review. Injury 37, S3–S14 (2006). 10.1016/j.injury.2006.04.003 16651069

[b42] Leslie D. C. . A bioinspired omniphobic surface coating on medical devices prevents thrombosis and biofouling. Nat. Biotechnol. 32, 1134–1140 (2014). 25306244 10.1038/nbt.3020

[b43] Ploux L. . Opposite Responses of Cells and Bacteria to Micro/Nanopatterned Surfaces Prepared by Pulsed Plasma Polymerization and UV-Irradiation. Langmuir 25, 8161–8169 (2009). 19518080 10.1021/la900457f

[b44] Huang Y. . The construction of hierarchical structure on Ti substrate with superior osteogenic activity and intrinsic antibacterial capability. Sci. Rep. 4, 6172 (2014). 25146099 10.1038/srep06172 PMC4141259

[b45] Oh S. . Stem cell fate dictated solely by altered nanotube dimension. Proc. Natl. Acad. Sci. 106, 2130–2135 (2009). 19179282 10.1073/pnas.0813200106 PMC2650120

[b46] Watari S. . Modulation of osteogenic differentiation in hMSCs cells by submicron topographically-patterned ridges and grooves. Biomaterials 33, 128–136 (2012). 21982295 10.1016/j.biomaterials.2011.09.058 PMC3208761

[b47] Kumar G. . The determination of stem cell fate by 3D scaffold structures through the control of cell shape. Biomaterials 32, 9188–9196 (2011). 21890197 10.1016/j.biomaterials.2011.08.054 PMC3428125

[b48] Chen W. . Nanoroughened Surfaces for Efficient Capture of Circulating Tumor Cells without Using Capture Antibodies. ACS Nano 7, 566–575 (2013). 23194329 10.1021/nn304719q PMC3962680

[b49] Kumar S., Raj S., Sarkar K. & Chatterjee K. Engineering a Multi-biofunctional Composite Using Poly(ethyleneimine) Decorated Graphene Oxide for Bone Tissue Regeneration. Nanoscale 8, 6820–6836 (2016). 26955801 10.1039/c5nr06906h

[b50] Asakura A., Rudnicki M. A. & Komaki M. Muscle satellite cells are multipotential stem cells that exhibit myogenic, osteogenic, and adipogenic differentiation. Differentiation 68, 245–253 (2001). 11776477 10.1046/j.1432-0436.2001.680412.x

[b51] Javed A. . Structural coupling of Smad and Runx2 for execution of the BMP2 osteogenic signal. Journal of Biological Chemistry 283, 8412–8422 (2008). 18204048 10.1074/jbc.M705578200 PMC2417186

[b52] Metavarayuth K., Sitasuwan P., Zhao X., Lin Y. & Wang Q. Influence of Surface Topographical Cues on the Differentiation of Mesenchymal Stem Cells *in Vitro*. ACS Biomater. Sci. Eng. 2, 142–151 (2016). 10.1021/acsbiomaterials.5b00377 33418629

[b53] McNamara L. E. . Skeletal stem cell physiology on functionally distinct titania nanotopographies. Biomaterials 32, 7403–7410 (2011). 21820172 10.1016/j.biomaterials.2011.06.063

[b54] Klymov A., Prodanov L., Lamers E., Jansen J. A. & Walboomers X. F. Understanding the role of nano-topography on the surface of a bone-implant. Biomat. Sci. 1, 135–151 (2013). 10.1039/c2bm00032f 32481794

[b55] Pogodin S. . Biophysical Model of Bacterial Cell Interactions with Nanopatterned Cicada Wing Surfaces. Biophys. J. 104, 835–840 (2013). 23442962 10.1016/j.bpj.2012.12.046 PMC3576530

[b56] Li X. Bactericidal mechanism of nanopatterned surfaces. Physical Chemistry Chemical Physics 18, 1311–1316 (2016). 26661138 10.1039/c5cp05646b

[b57] Li X. & Chen T. Enhancement and suppression effects of a nanopatterned surface on bacterial adhesion. Physical Review E 93, 052419 (2016). 27300935 10.1103/PhysRevE.93.052419

[b58] Nikolaev N. I., Muller T., Williams D. J. & Liu Y. Changes in the stiffness of human mesenchymal stem cells with the progress of cell death as measured by atomic force microscopy. J. Biomech. 47, 625–630 (2014). 24373509 10.1016/j.jbiomech.2013.12.004

[b59] Tuson H. H. . Measuring the stiffness of bacterial cells from growth rates in hydrogels of tunable elasticity. Mol. Microbiol. 84, 874–891 (2012). 22548341 10.1111/j.1365-2958.2012.08063.x PMC3359400

[b60] Fukuda A. . Osteoinduction of porous Ti implants with a channel structure fabricated by selective laser melting. Acta Biomater. 7, 2327–2336 (2011). 21295166 10.1016/j.actbio.2011.01.037

[b61] Ripamonti U., Crooks J., Khoali L. & Roden L. The induction of bone formation by coral-derived calcium carbonate/hydroxyapatite constructs. Biomaterials 30, 1428–1439 (2009). 19081131 10.1016/j.biomaterials.2008.10.065

[b62] Yuan H. . A preliminary study on osteoinduction of two kinds of calcium phosphate ceramics. Biomaterials 20, 1799–1806 (1999). 10509190 10.1016/s0142-9612(99)00075-7

[b63] Ripamonti U., Roden L. C. & Renton L. F. Osteoinductive hydroxyapatite-coated titanium implants. Biomaterials 33, 3813–3823 (2012). 22364700 10.1016/j.biomaterials.2012.01.050

[b64] Bahl S., Suwas S. & Chatterjee K. The importance of crystallographic texture in the use of titanium as an orthopedic biomaterial. RSC Adv. 4, 38078–38087 (2014).

[b65] Kumar S. . Chemical functionalization of graphene to augment stem cell osteogenesis and inhibit biofilm formation on polymer composites for orthopedic applications. ACS Appl. Mater. Interfaces 7, 3237–3252 (2015). 25584679 10.1021/am5079732

