# Supplementary material for: Nanoscale Topography on Black Titanium Imparts Multi-biofunctional Properties for Orthopedic Applications
Source: Sci Rep. 2017 Jan 23;7:41118. doi: 10.1038/srep41118 (PMC5253769; doi:10.1038/srep41118)
Supplement: Supplementary Information [file srep41118-s1.pdf]

***Supplementary Information for***

# Nanoscale Topography on Black Titanium Imparts Multi-biofunctional Properties for Orthopedic Applications

Jafar Hasan[1], Shubham Jain[1], Kaushik Chatterjee[1]*

[1]Department of Materials Engineering

Indian Institute of Science, Bangalore 560012 India

[*] *author to whom all correspondence should be addressed:*

Email: kchatterjee@materials.iisc.ernet.in; Tel: +91-80-22933408

**Table S1.** Roughness analysis of the titanium surfaces measured by AFM

| Etching time | **0 min*** | **5 min** | **10 min** | **20 min** |
|---|---|---|---|---|
| ***$R_q$* (nm)** | 8 ± 1 | 72 ± 2 | 227 ± 8 | 262 ± 9 |
| ***$R_a$* (nm)** | 5 ± 1 | 57 ± 1 | 186 ± 6 | 215 ± 9 |
| **Surface area ($\mu m^2$)** | 100 ± 1 | 142 ± 1 | 326 ± 27 | 362 ± 40 |
| **Projected area ($\mu m^2$)** | 100 ± 1 | 100 ± 1 | 100 ± 1 | 100 ± 1 |
| ***$R_{max}$* (nm)** | 143 ± 19 | 586 ± 33 | 1511 ± 23 | 1728 ± 110 |
| **Skewness (nm)** | 0.1 ± 1.4 | 0.3 ± 0.1 | - 0.1 ± 0.1 | - 0.1 ± 0.1 |

* unetched smooth control surface

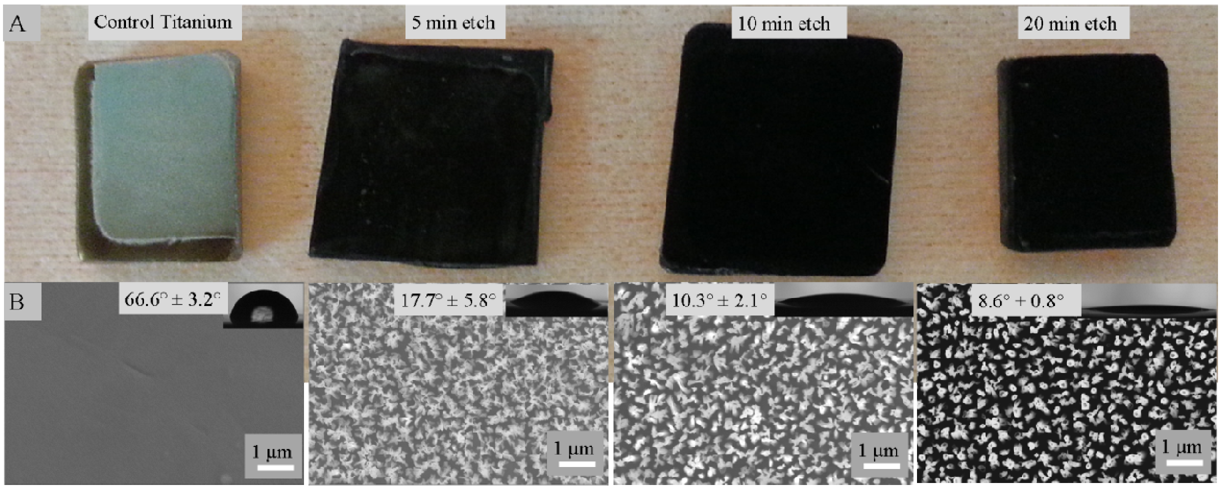


**Figure S1.** A, Photographs (top) and B, SEM (bottom) of control and black titanium etched for 5, 10 and 20 minutes with insets showing representative photographs of a water droplet on these surfaces.

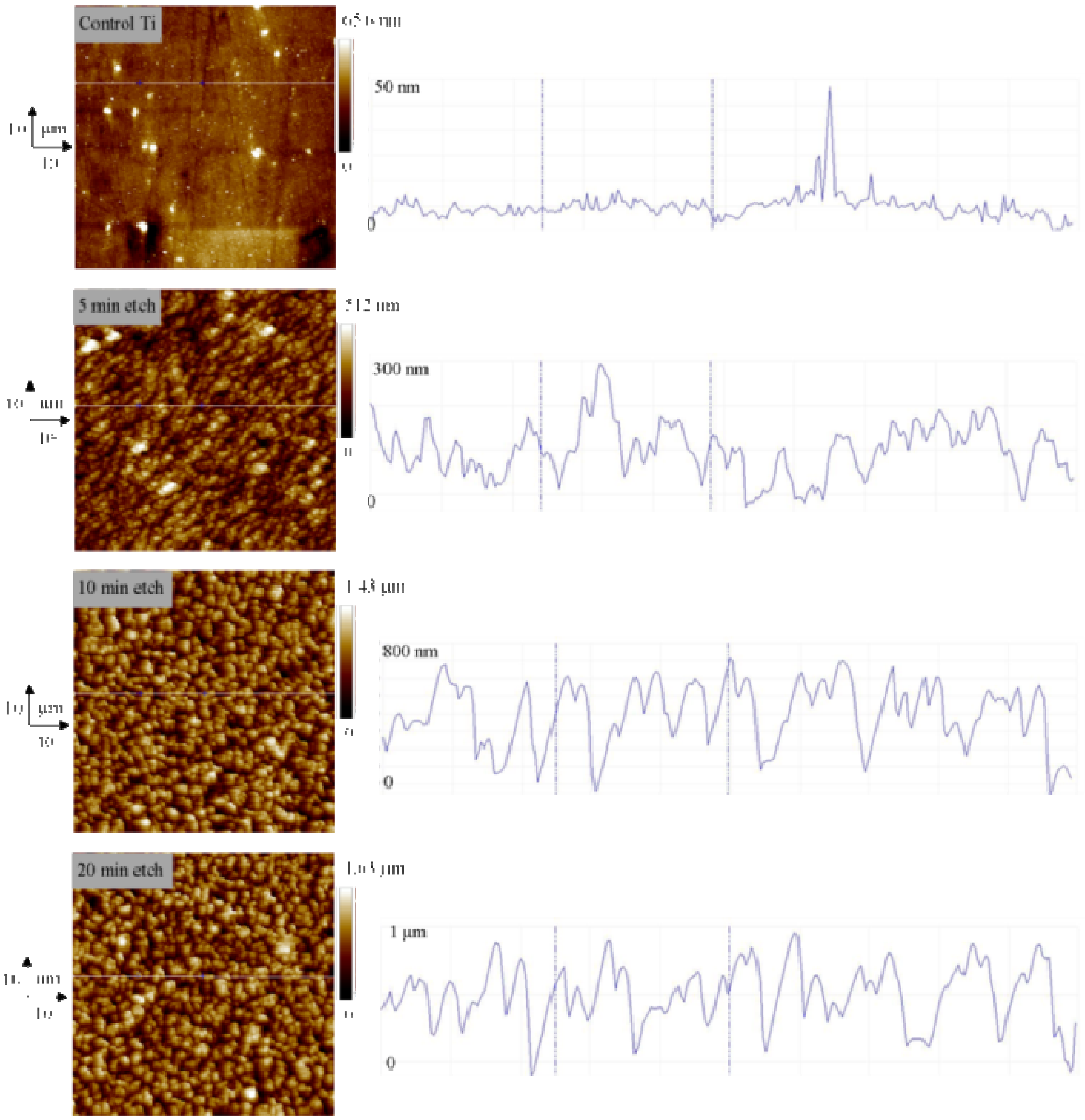


**Figure S2.** AFM data of control and 5, 10 and 20 minute etched black titanium samples. Representative micrographs are presented on the left and the height profiles on the right.

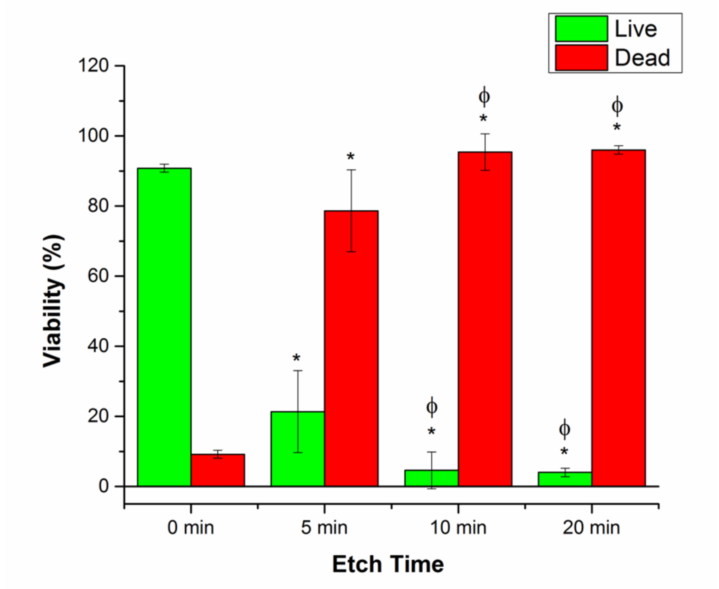


**Figure S3** Plot of viability of *E. coli* showing that the 10 minute etched sample is the minimum etching time for maximum bactericidal activity. Statistically significant difference ($p < 0.05$) with respect to the control (0 min) and 5 min etched titanium are indicated by * and ϕ respectively.

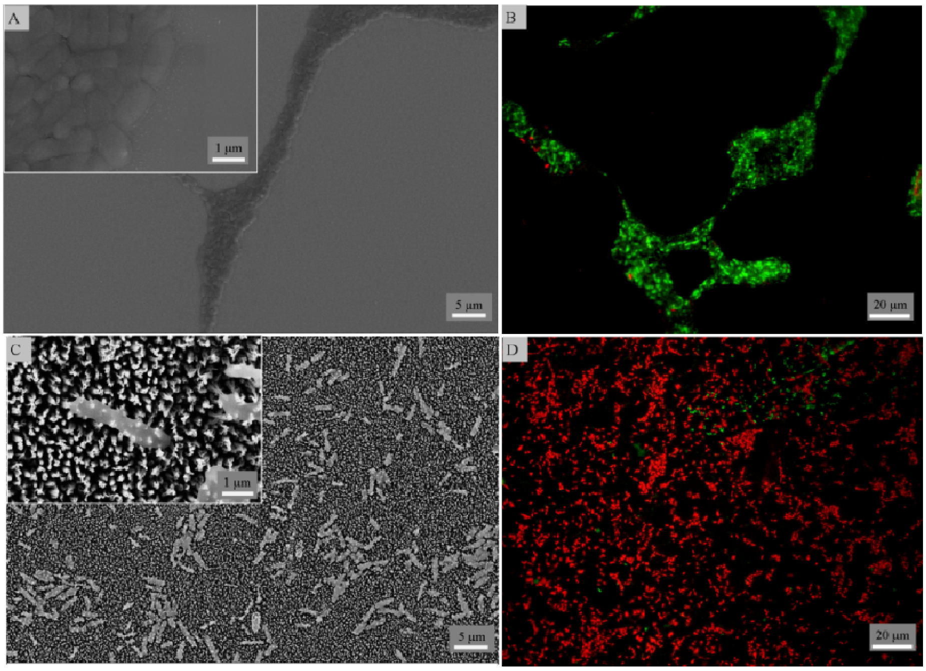


**Figure S4.** (A, C) SEM and (B, D) fluorescent microscopy images of *E. coli* attachment on control (top) and the 10 minute etched black titanium (bottom) surfaces.

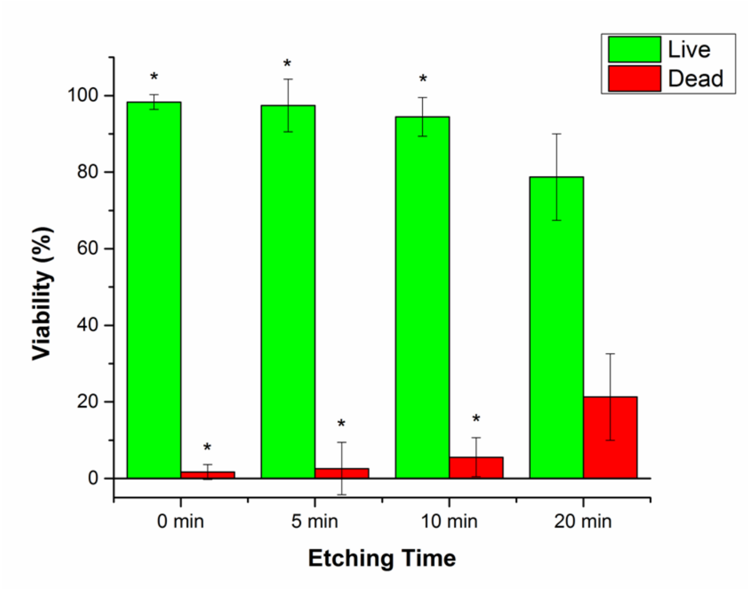


**Figure S5.** Plot of hMSC viability showing that 10 min etched sample is the maximal time of etching that does not compromise cell viability. Statistically significant differences ($p < 0.05$) in the control (0 min), 5 min, 10 min and 20 min etched titanium is indicated by *.

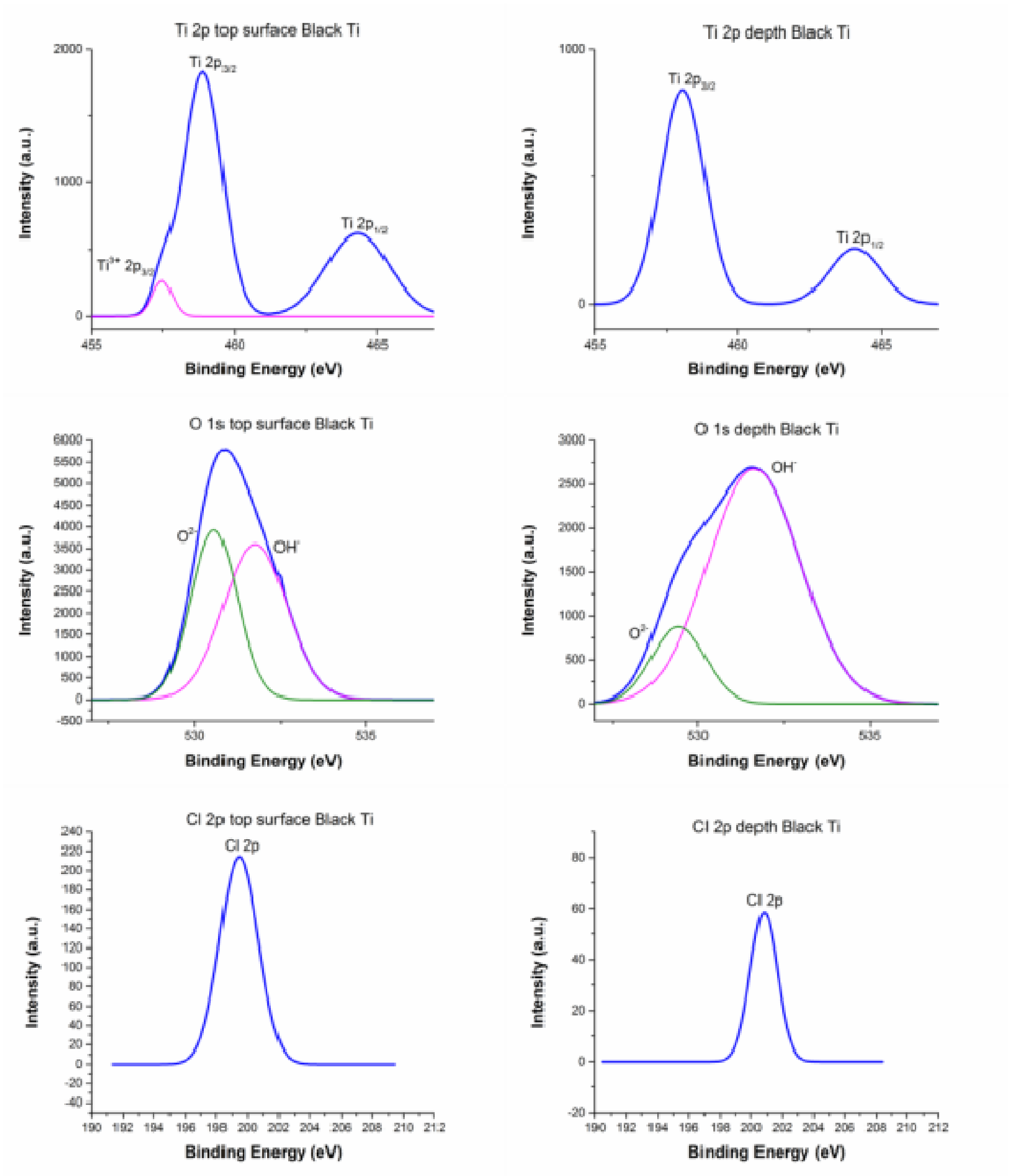


**Figure S6.** High resolution XPS spectra of black titanium before (left) and after (right) destructive etching on the sample in order to determine the chemical composition on the top layer and the interior of the nanostructured surface.

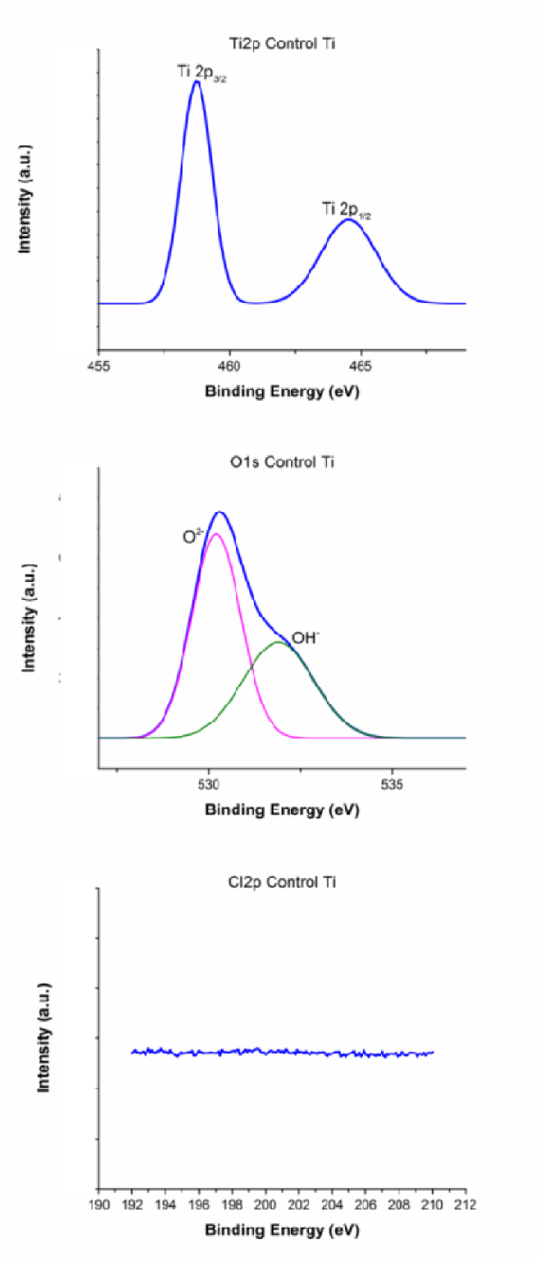


**Figure S7.** XPS data of the control titanium surface

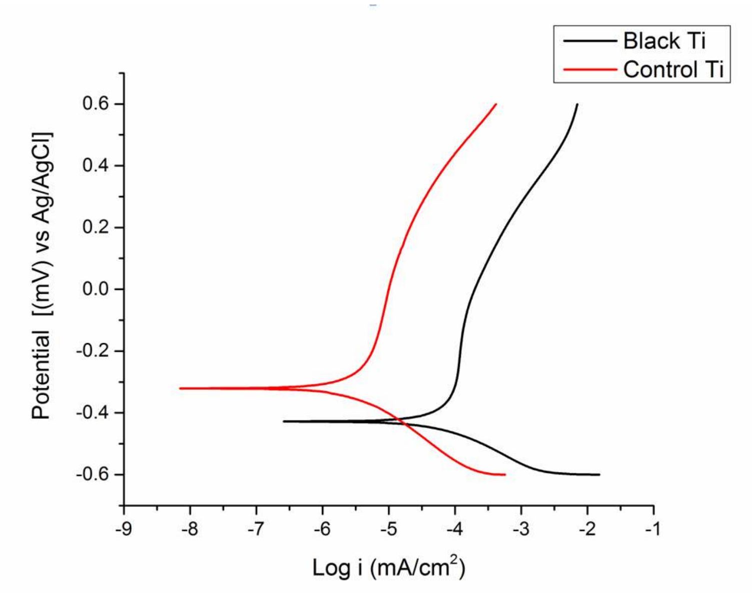


**Figure S8.** Potentiodynamic polarization curves of control and black titanium samples measured in 0.9 % NaCl solution.

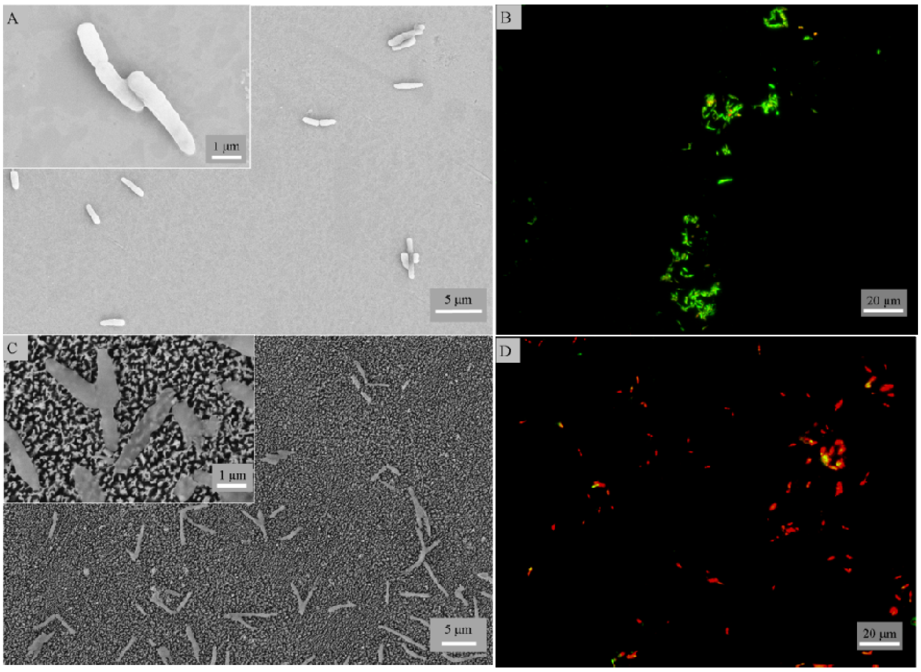


**Figure S9.** (A, C) SEM and (B, D) fluorescent microscopy images of *M. smegmatis* attachment on control (top) and the black titanium (bottom) surfaces.

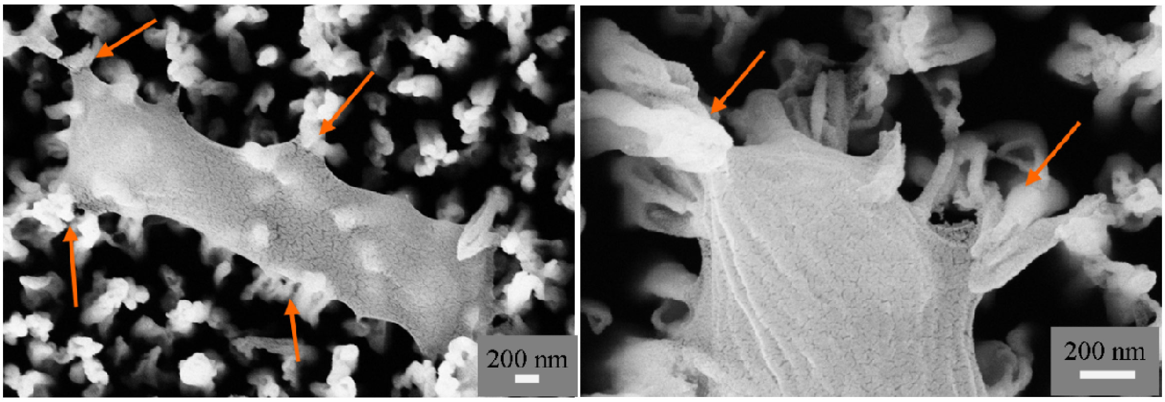


**Figure S10**. High magnification SEM images of the interaction of *P. aeruginosa* cells on the black titanium surface.

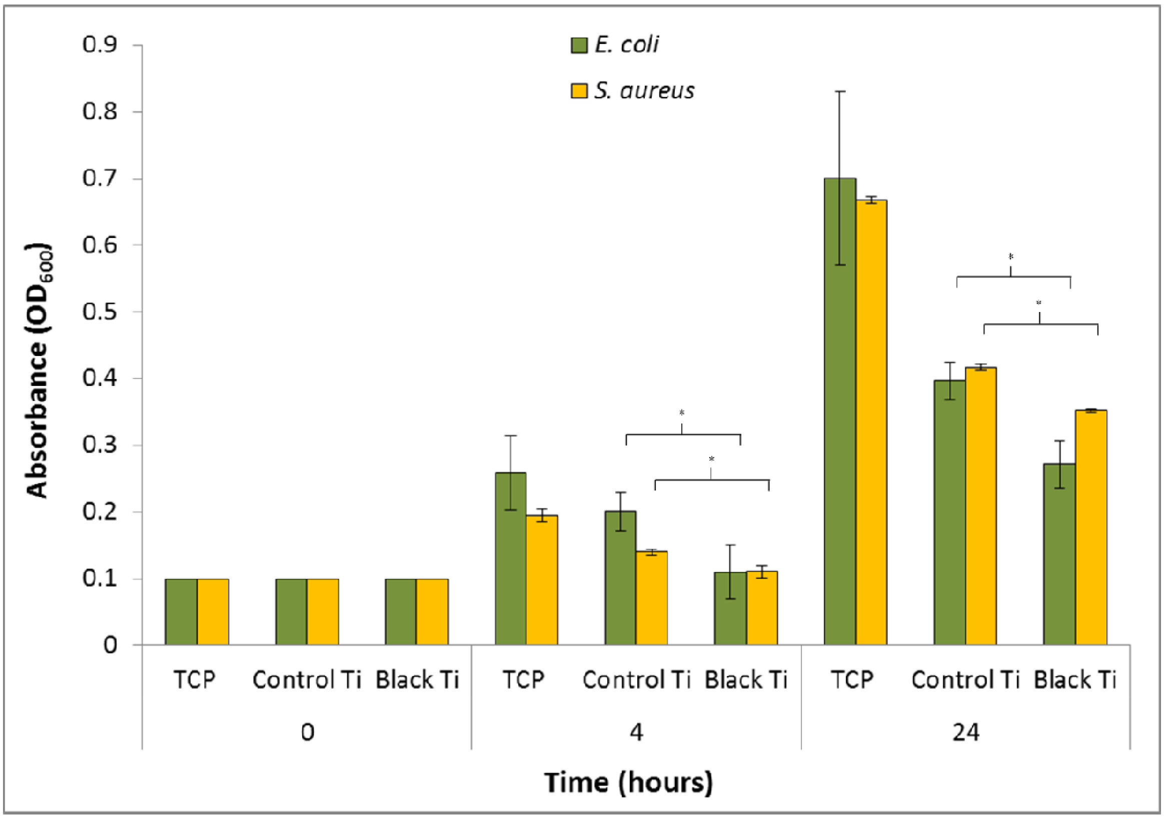


**Figure S11**. Absorbance values of the bacterial suspension of *E. coli* and *S. aureus* on TCP plate, control titanium and black titanium samples measured on a 48-well plate at three time intervals.

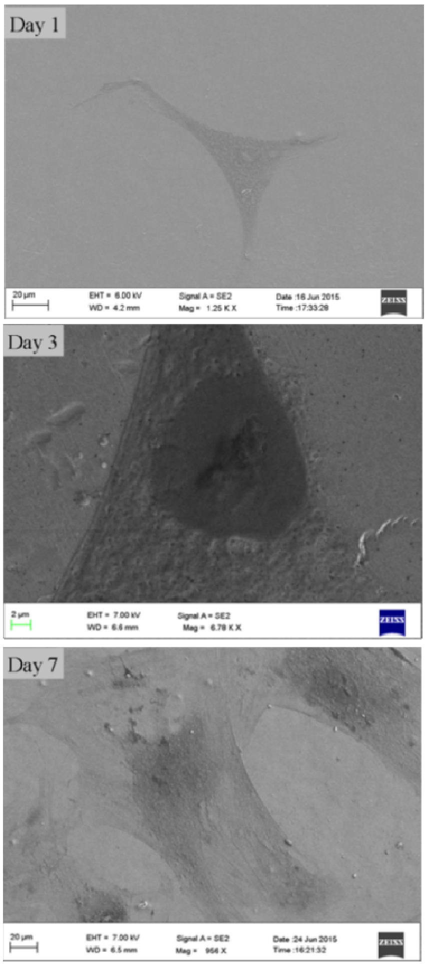


**Figure S12**. SEM micrographs displaying hMSCs attached on control titanium at days 1, 3 and 7.

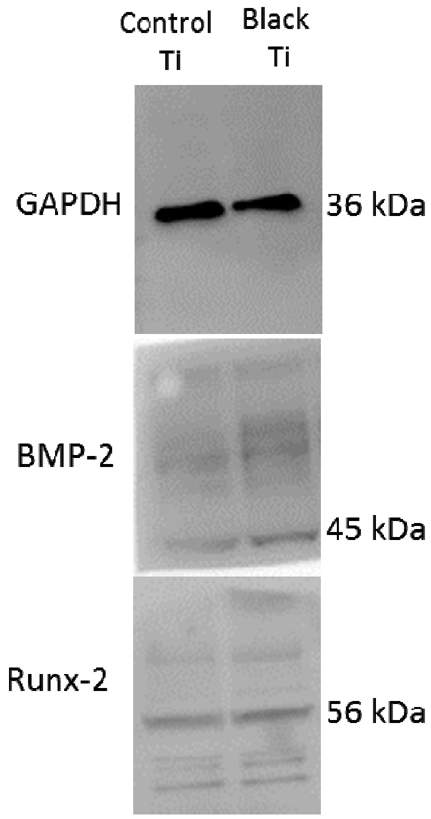


**Figure S13.** Full length gels of the protein expressions of the control and black titanium samples.
